# Syk and IRAK1 Contribute to Immunopharmacological Activities of Anthraquinone-2-carboxlic Acid

**DOI:** 10.3390/molecules21060809

**Published:** 2016-06-22

**Authors:** Jae Gwang Park, Young-Jin Son, Mi-Yeon Kim, Jae Youl Cho

**Affiliations:** 1 Department of Genetic Engineering, Sungkyunkwan University, Suwon 16419, Korea; wannabejk@naver.com; 2 Department of Pharmacy, Sunchon National University, Suncheon 57922, Korea; sony@sunchon.ac.kr; 3 School of Systems Biomedical Science, Soongsil University, Seoul 06978, Korea

**Keywords:** anthraquinones, anthraquinone-2-carboxylic acid, inflammatory response, IRAK1

## Abstract

Anthraquinone-2-carboxlic acid (9,10-dihydro-9,10-dioxo-2-anthracenecarboxylic acid, AQCA) was identified as one of the major anthraquinones in Brazilian taheebo. Since there was no report explaining its immunopharmacological actions, in this study, we aimed to investigate the molecular mechanism of AQCA-mediated anti-inflammatory activity using reporter gene assays, kinase assays, immunoblot analyses, and overexpression strategies with lipopolysaccharide (LPS)-treated macrophages. AQCA was found to suppress the release of nitric oxide (NO) and prostaglandin (PG) E_2_ from LPS-treated peritoneal macrophages without displaying any toxic side effects. Molecular analysis revealed that AQCA was able to inhibit the activation of the nuclear factor (NF)-κB and activator protein (AP)-1 pathways by direct suppression of upstream signaling enzymes including interleukin-1 receptor-associated kinase 1 (IRAK1) and spleen tyrosine kinase (Syk). Therefore, our data strongly suggest that AQCA-mediated suppression of inflammatory responses could be managed by a direct interference of signaling cascades including IRAK and Syk, linked to the activation of NF-κB and AP-1.

## 1. Introduction

Chronic inflammation is a primary cause of several diseases such as asthma, peptic ulcer, rheumatoid arthritis, hepatitis, and some cancers [1,2]. Chronic inflammation leads to overproduction of oxygen free radicals and pro-inflammatory cytokines, resulting in excessive infiltration of various immune cells. This causes tissue damage and organ dysfunction [3,4]. In addition, diseases accompanied by severe inflammation show increased mortality rate compared with those accompanied by mild inflammation [5,6]. Therefore, the generation and control of chronic inflammation is an important medical issue. 

People have traditionally used medicinal plants or nutraceuticals such as basil, chamomile, parsley, ginseng, and taheebo to cure inflammatory diseases [7,8]. Recently, researchers have identified many beneficial nutrients such as vitamins, terpenoids, flavonoid, saponins, alkaloids, and quinones in these foods, and such compounds were shown to ameliorate immune disorders by controlling oxidative and inflammatory factors [9,10,11,12,13,14]. In addition to these components, anthraquinone compounds are considered as another useful constituent, presented in a number of medicinal plants, and therefore have potential clinical applications. Many kinds of anthraquinone compounds have been identified, and the antioxidative, anti-asthmatic and anti-inflammatory effects of some of these compounds, such as emodin and rhein, are well established [15,16]. However, the precise mechanism underlying the pharmaceutical activity of other anthraquinones has not been established.

Previously, we have found that anthraquinone-2-carboxylic acid (AQCA), a representative anthraquinone-type compound derived from taheebo is able to relieve symptoms of inflammatory diseases and severe pains at 3 and 30 mg/kg [17]. Since exact anti-inflammatory mechanism of AQCA has not been proposed yet, in the present study, we aimed to understand the molecular mechanism of AQCA. To do this, direct target molecule(s) involved in the anti-inflammatory response of AQCA was identified using reporter gene assays, kinase assays, immunoblot analysis, and overexpression strategies.

## 2. Results

### 2.1. Effect of AQCA on Inflammatory Responses of Macrophages Induced by LPS

**Figure 1:**
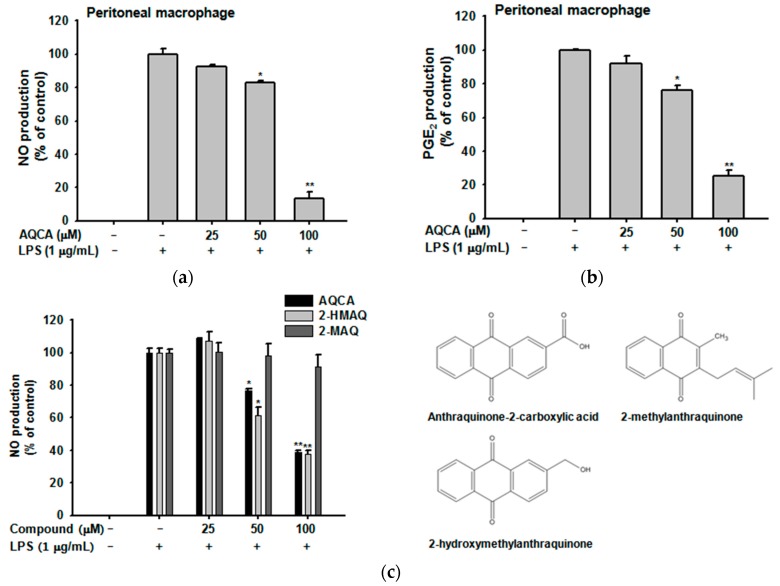

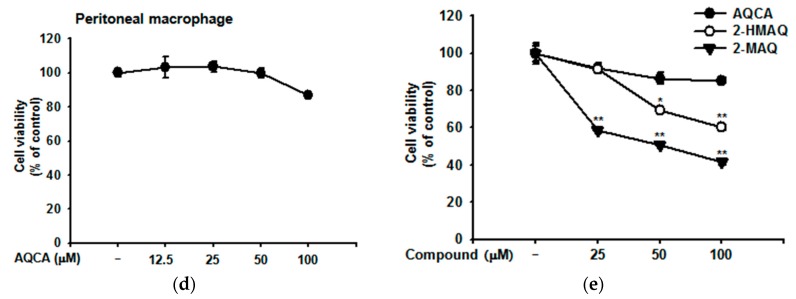
Effect of anthraquinone-2-carboxlic acid (AQCA) on the production of nitric oxide (NO) and prostaglandine E_2_ (PGE_2_) in macrophages and cell viability. Peritoneal macrophages (2 × 10^6^ cells/mL) or RAW264.7 cells (1 × 10^6^ cells/mL) were treated with lipopolysaccharide (LPS) (1 μg/mL) in the presence or absence of AQCA, 2-hydroxymethylanthraquinone (2-HMAQ), or 2-MAQ for 24 h. (**a**–**c**–left panel) Concentrations of NO or PGE_2_ in the culture supernatants were determined using the Griess assay and enzyme immunoassay (EIA); (**c**–right panel) Chemical structures of AQCA, 2-HMAQ, and 2-MAQ; (**d**–**e**) Peritoneal macrophages or RAW264.7 cells were treated with AQCA (0 to 100 μM) for 24 h, respectively. Cell viability was evaluated using the (3-4,5-dimethylthiazol-2-yl)-2,5-diphenyltetrazolium bromide, tetrazole (MTT) assay. All data are expressed as the mean ± SD of experiments performed with six samples. Similar inhibitory patterns were observed in two repeat experiments. * *p* < 0.05 and ** *p* < 0.01 compared to control (LPS alone) or normal (without LPS) groups.

As shown in Figure 1a, AQCA suppressed the production of nitric oxide in LPS-activated peritoneal macrophages in a dose-dependent manner. Moreover, prostaglandine E_2_ (PGE_2_) production was blocked in a similar manner (Figure 1b). AQCA inhibited the release of NO from RAW264.7 cells during LPS stimulation to a similar extent as the structurally similar 2-hydroxymethylanthraquinone (2-HMAQ), whereas 2-methylanthra-quinone (2-MAQ) had no effect (Figure 1c). AQCA did not significantly affect the viability of peritoneal macrophages (Figure 1d) or RAW264.7 cells (Figure 1e). In contrast, 2-HMAQ and 2-MAQ, two other anthraquinones extracted from taheebo derivatives, suppressed the viability of RAW264.7 cells under unstimulated conditions (Figure 1e).

### 2.2. Effect of AQCA on Transcriptional Activation of the Inflammatory Response

**Figure 2:**
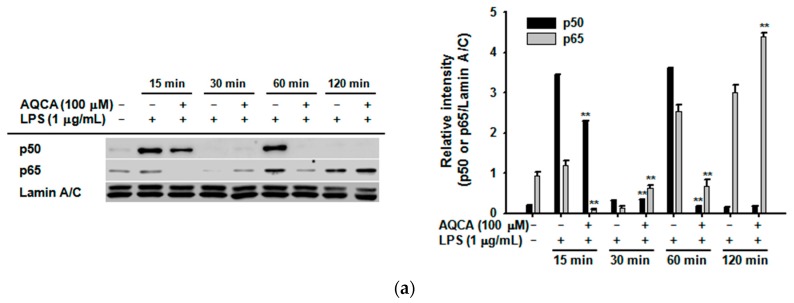

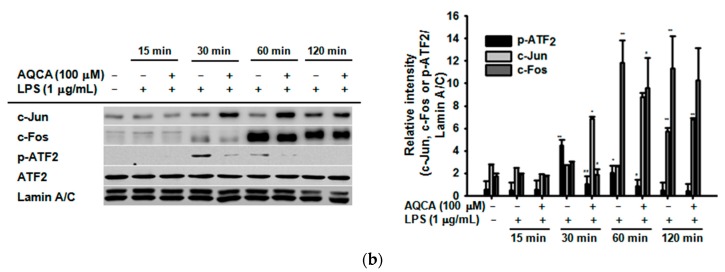
Effect of AQCA on transcriptional activation of the inflammatory response. (**a**,**b**) nuclear fractions were prepared from LPS-treated RAW264.7 cells and the total protein levels of p50, p60, c-Jun, c-Fos, p-ATF2, ATF2, and Lamin A/C were analyzed by immunoblot analyses. Intensity relative to Lamin A/C level was calculated using the DNR Bio-Imaging system (**a** right panel and **b** right panel). * *p* < 0.05 and ** *p* < 0.01 compared with the control (LPS alone) group.

To define the precise role of AQCA in the inflammatory response, we examined whether the production of inflammatory mediators was affected by AQCA at the transcriptional level. Likewise, the nuclear translocation levels of p50 and p65 at 15 and 60 min (Figure 2a) and c-Fos and phospho-ATF2 at 30 and 60 min (Figure 2b) were also significantly downregulated by AQCA in LPS-induced RAW264.7 cells. However, the nuclear translocation of c-Jun was not reduced by AQCA exposure, rather it was enhanced at 30 and 60 min.

### 2.3. Effect of AQCA on the Activation of Upstream Enzymes for NF-κB and AP-1 Activation

**Figure 3:**
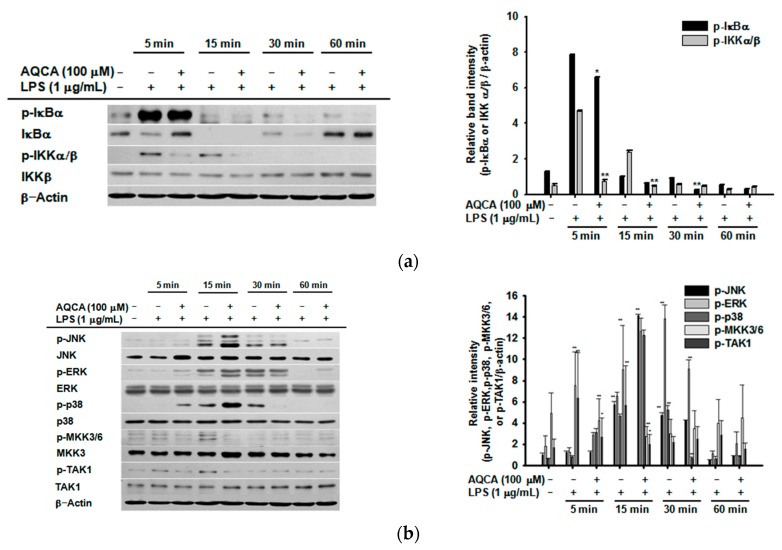

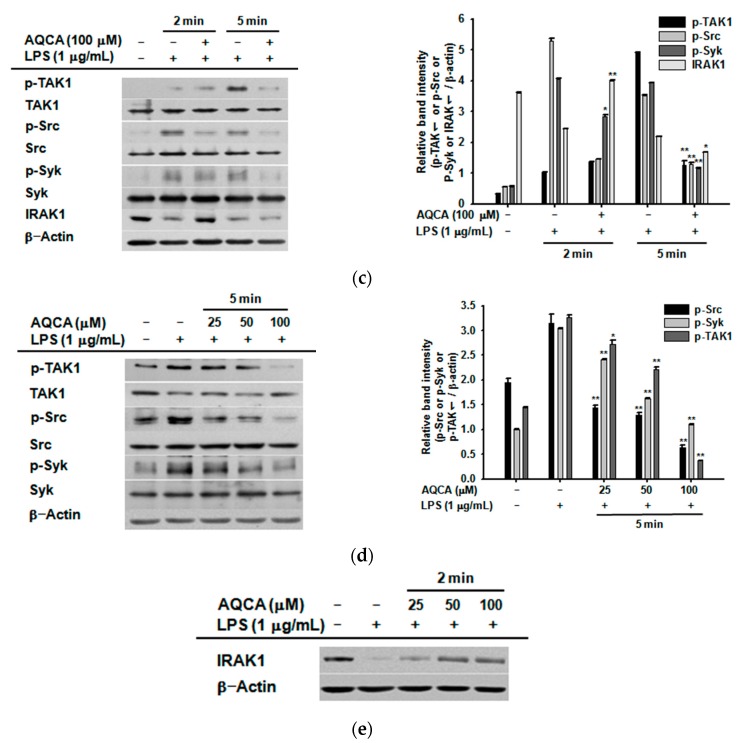
Effect of AQCA on nuclear factor (NF)-κB and activator protein (AP)-1 activation signaling. (Left panels of **a**–**e**) RAW264.7 cells (5 × 10^6^ cells/mL) were incubated with LPS (1 μg/mL) in the presence or absence of AQCA (25, 50, or 100 μM) for the indicated times. The levels of total or phosphorylated IκBα, IκB kinase α/β (IKKα/β), IKKβ, protein kinase B (AKT), transforming growth factor β-activated kinase 1 (TAK1), spleen tyrosine kinase (Syk), Src, interleukin-1 receptor-associated kinase 4 (IRAK1), c-Jun N-terminal kinase (JNK), extracellular signal–regulated kinase (ERK), p38, mitogen-activated protein kinase kinase 3/6 (MKK3/6), and β-actin in whole cell lysates were identified using immunoblot analyses. Relative intensity (right panels of **a**–**d**) was calculated relative to β-actin levels using the DNR Bio-Imaging system. All data (right panels of **a**–**d**) are expressed as the mean ± SD of experiments performed with three samples. * *p* < 0.05 and ** *p* < 0.01 compared to the control (LPS alone) group.

Upstream inhibitory target molecules of AQCA involved in nuclear factor (NF)-κB and activator protein (AP)-1 activation were identified in LPS-treated RAW264.7 cells. The phosphorylation of IκBα at 15, 30, and 60 min and of IκB kinase (IKK) at 5 and 15 min in LPS-treated RAW264.7 cells was decreased by AQCA (100 μM) (Figure 3a). The phosphorylation of p38 at 30 min, c-Jun N-terminal kinase (JNK) at 30 min, mitogen-activated protein kinase kinase 3/6 (MKK3/6) at 5 and 15 min, and transforming growth factor β-activated kinase 1 (TAK1) at 5 and 15 min was also reduced by AQCA, while there was no inhibition of extracellular signal–regulated kinase (ERK) phosphorylation (Figure 3b). At earlier time points, phosphorylation of their upstream enzymes, such as Src, Syk, and TAK1, and degradation of IRAK1 were also decreased by AQCA in a time-dependent (2 and 5 min) and dose-dependent (25–100 μM) manner (Figure 3c–e).

### 2.4. Effect of AQCA on the Activity of Upstream Enzymes

**Figure 4:**
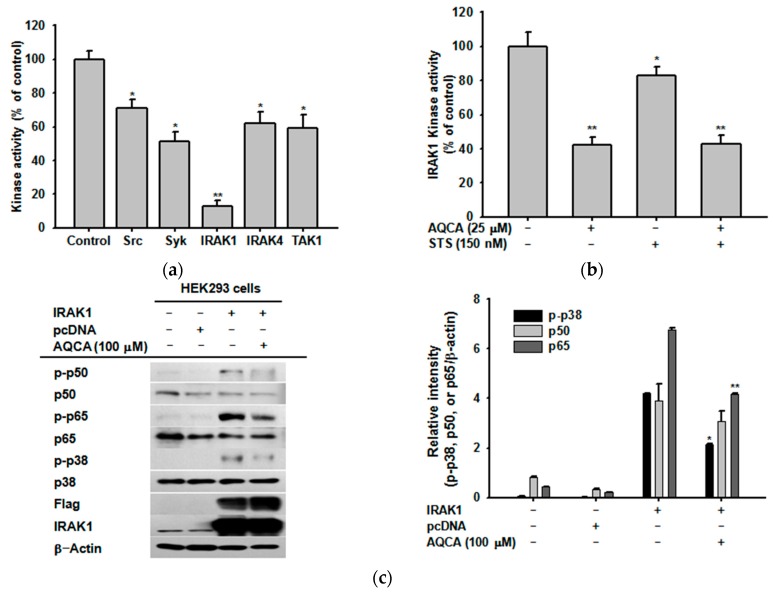
Effect of AQCA on IRAK1 enzyme activity. (**a**,**b**) the inhibitory effect of AQCA (50 μM) or staurosporin (STS) (150 nM) on the enzyme activity of Src, Syk, IRAK1, IRAK4, or TAK1 was determined using a conventional kinase assay with purified enzymes provided from Millipore (Billerica, MA, USA). The control was set as 100% activity for each enzyme with vehicle treatment only. (**c** left panel) HEK293 cells transfected with IRAK1 (1 μg/mL) for 36 h were treated with AQCA (100 μM) for 12 h. The levels of total or phosphorylated p50, p65, p38, IRAK1, Flag, and β-actin in whole cell lysates were identified using immunoblot analyses. Relative intensity (**c** right panel) was calculated relative to β-actin levels using the DNR Bio-Imaging system. All data (**a**–**c** left panel) are expressed as the mean ± SD of experiments performed with three samples. * *p* < 0.05 and ** *p* < 0.01 compared to the control (purified enzyme alone or Flag-IRAK1 alone) groups.

To confirm whether inhibition of the phosphorylation and degradation of Src, Syk, IRAK1, IRAK4, and TAK1 was directly mediated by AQCA, we performed a conventional kinase assay with purified enzymes. Of the enzymes tested, the activity of IRAK1 was the most potently suppressed by AQCA, whereas the other enzymes (Syk, Src, IRAK4, and TAK1) were partially suppressed by 30% to 50% (Figure 4a). As expected, AQCA (60% inhibition at 25 μM) competed for ATP when treated in combination with the ATP-binding site-targeting kinase inhibitor staurosporin (STS) (20% inhibition at 150 nM) (Figure 4b). To validate IRAK1 as the strongest target, we employed a transfection strategy with the *IRAK1* gene using HEK293 cells. As shown in Figure 4c, overexpression of *IRAK1* for 36 h strongly increased the levels of phosphorylated p50, p65, and p38. As expected, AQCA treatment for 12 h suppressed phosphorylation of these proteins without altering Flag and IRAK1 protein levels (Figure 4c).

## 3. Discussion

Our previous *in vivo* tests performed with AQCA (3 and 30 mg/kg) orally administered in aspirin-induced gastritis, arachidonic acid-triggered ear edema, and acetic acid-induced writhing mouse models [17] strongly suggested that AQCA has potential as an oral anti-inflammatory drug. Consistent with our findings, other anthraquinone-type compounds show similar properties. For example, orally administered emodin (3-methyl-1,6,8-trihydroxyanthraquinone), one of the active components of the root and rhizome of *Rheum palmatum* that has been used for more than 2000 years in China, was found to exert inhibitory activities on metabolic disorders in diet-induced obese mice [18]. Rhein (4,5-dihydroxyanthraquinone-2-carboxylic acid), another major anthraquinone compound from the same plant, has been demonstrated to ameliorate hepatic steatosis associated with nonalcoholic fatty liver disease [19]. In addition, numerous medicinal plants with anthraquinone-type ingredients are known to display anti-gastritis, anti-hepatitis, and anti-arthritis properties [20]. Thus, our previous data and these reports indicate that anthraquinones might show effective anti-inflammatory activity and our data further suggest AQCA as a candidate compound for development as a novel immunosuppressant.

To understand the molecular mechanism of *in vivo* anti-inflammatory activity of AQCA, we employed *in vitro* models with RAW264.7 cells and peritoneal macrophages through biochemical and molecular biological approaches. The observation that the production of NO and PGE_2_ from LPS-treated peritoneal macrophages and RAW264.7 cells was inhibited by AQCA (Figure 1a–c) implies that suppression of macrophage activation might be one of the critical factors in AQCA-mediated anti-inflammatory action. In fact, our findings that both NF-κB and AP-1 activities (as assessed by luciferase assays [21]), were significantly reduced by AQCA [17], and that nuclear translocation of p50/p65 and c-Fos/p-ATF2 at certain time points was reduced by AQCA in a time-dependent manner (Figure 2b,c), led us to assume that one or multiple enzymes involved in the signaling pathways for NF-κB and AP-1 activation might be targeted by this compound.

Accumulated results from our laboratory suggest that Src, Syk, IRAK1, IRAK4, ERK, p38, and JNK are important upstream signaling enzymes for NF-κB and AP-1 activation in LPS-treated macrophages [22,23,24,25]. On the basis of this information, we investigated whether AQCA regulated the activation of upstream inflammatory signaling enzymes. As expected, the activation of p38, Src, Syk, and IRAK1 in LPS-treated RAW264.7 cells, as assessed by phosphorylation and degradation, was suppressed by AQCA, suggesting that these enzymes might be targeted in the anti-inflammatory activity of AQCA. Especially, the inhibitory potency of AQCA against the kinase activity of IRAK1, alone and in combination treatment with the ATP-binding inhibitor STS, strongly indicates that IRAK1 is the most strongly inhibited target and that the ATP binding site of this enzyme might be the AQCA-accessible site. In agreement with this, it was previously reported that inhibitors of p38 (SB203580), Src (PP2), and Syk (piceatannol) suppressed the expression of *TNF-*α, *iNOS*, and *COX-2*, similar to the response to AQCA [17]. In addition, numerous reports have suggested the role of p38, Src, Syk, and IRAK1 in NF-κB and AP-1 activation [21,26,27,28]. Early IκBα phosphorylation (within 1 h) in LPS-treated RAW264.7 cells associated with early activation of NF-κB is tightly linked to Syk phosphorylation [29]. It was previously reported that p38 is a positive player in the regulation of macrophage-mediated inflammation via activation of AP-1 [27]. Src, a proto-oncogene involved in cell survival and proliferation [30], is regarded as a strong activator of NF-κB induction by pro-oxidants through TLR4 signaling [28]. Additionally, early rearrangement of the actin cytoskeleton under LPS stimulation is linked to the activation of NF-κB via upregulation of Src activity [31]. IRAK1 was also revealed to stimulate both NF-κB and AP-1 pathways in LPS-stimulated macrophages [32]. Moreover, it has been shown that the activation and translocation of NF-κB or AP-1 are time-dependently controlled by upstream signaling [29]. 

Distinctively, translocation levels of p65 at 30 and 120 min and c-Jun at 30, 60 and 120 min were enhanced by AQCA (Figure 2c), implying not only that upstream enzymes regulating the translocation of these transcription factors at these time points are not targeted but also that suppression of other transcription factors by this compound might be enough to display its anti-inflammatory activity. Such increased activity of c-Jun and p65 might be result in the activation of JNK1/2 and p38 at 15 min, ERK at 60 min under AQCA treatment conditions (Figure 3b). Indeed, these enzymes were reported to affect the activation of NF-κB and AP-1 in human melanoma and HCT116 cells activated by UV irradiation [33,34]. These results suggest that AQCA might be a useful drug to study time-dependent activation of inflammation-regulatory transcription factors. Whether the activation of these enzymes by AQCA is directly linked to the activation of c-Jun and p65 will be further examined. Taken together, these findings clearly suggest that suppression of IRAK1 and Syk by AQCA predominantly contributes to its anti-inflammatory action.

## 4. Materials and Methods

### 4.1. Materials 

Anthraquinone-2-carboxylic acid (AQCA, Figure 1c right panel), 2-hydroxymethylanthra-quinone (2-HMAQ, Figure 1c right panel), 2-methylanthraquinone (2-MAQ, Figure 1c right panel), phorbol-12-myristate (PMA), and lipopolysaccharide (LPS, *E. coli* 0111:B4) were purchased from Sigma Chemical Co. (St. Louis, MO, USA). All other chemicals used in this study were of analytical grade from Sigma. Phospho-specific or total antibodies against Src, spleen tyrosine kinase (Syk), p38, interleukin-1 receptor-associated kinase 4 (IRAK4), and β-actin were obtained from Cell Signaling Technology (Beverly, MA, USA). RAW264.7 and HEK293 cells were purchased from the American Type Culture Collection (Manassas, VA, USA). The IRAK1 construct for the overexpression experiment was provided by Professor Michael Martin (Justus-Liebig University Giessen, Giessen, Germany) and Addgene (Cambridge, MA, USA).

### 4.2. Preparation of Peritoneal Macrophages

Six-week-old male C57BL/6 mice were purchased from B & K (Fremont, CA, USA). The mice had access to food pellets (Samyang, Daejeon, Korea) and water ad libitum and were housed under a 12-h light/12-h dark cycle. All studies were performed in accordance with the guidelines established by the Sungkyunkwan University Institutional Animal Care and Use Committee (Seoul, Korea). Peritoneal exudates were obtained from the mice (7 to 8 weeks old, 17 to 21 g) by lavage 4 days after intraperitoneal (i.p.) injection of 1 mL sterile 4% thioglycollate broth (Difco Laboratories, Detroit, MI, USA), as previously reported [35]. The exudates were washed with Roswell Park Memorial Institute (RPMI) 1640 medium containing 2% fetal bovine serum (FBS), and peritoneal macrophages (1 × 10^6^ cells/mL) were plated in 100-mm tissue culture dishes and incubated for 4 h at 37 °C in a 5% CO_2_ humidified atmosphere. 

### 4.3. Cell Culture and Drug Preparation

Murine macrophage RAW264.7 cells, peritoneal macrophages, and human embryonic kidney 293 (HEK293) cells were maintained in RPMI 1640 medium supplemented with 100 U/mL penicillin, 100 μg/mL streptomycin, and 10% FBS. Cells were grown at 37 °C and 5% CO_2_ in humidified air. AQCA compound was dissolved in 100% dimethlysulfoxide (DMSO).

### 4.4. Determination of Nitric Oxide (NO) and Prostaglandin E_2_ (PGE_2_) Production 

After preincubation of RAW264.7 cells or peritoneal macrophages (1 × 10^6^ cells/mL) for 18 h, the cells were treated with AQCA (0–100 μM) for 30 min and then further incubated with LPS (1 μg/mL) for 6 h (PGE_2_) or 24 h (NO). The inhibitory effects of AQCA on the production of NO and PGE_2_ were determined by analyzing the levels of these compounds using Griess reagents and enzyme immunoassay (EIA) kits (Cayman, Ann Abor, MI, USA), respectively, as previously described [36,37].

### 4.5. Cell Viability Assay

After preincubation of RAW264.7 cells (1 × 10^6^ cells/mL) for 18 h, AQCA (0–100 μM) was added and the cell suspensions were incubated for a further 24 h. The cytotoxic effects of AQCA were evaluated using a conventional (3-4,5-dimethylthiazol-2-yl)-2,5-diphenyltetrazolium bromide, tetrazole (MTT) assay as previously reported [36,38]. Three hours prior to culture termination, 10 μL of MTT solution (10 mg/mL in PBS, pH 7.4) was added and the cells were continuously cultured until termination of the experiment by the addition of 15% sodium dodecyl sulfate (SDS) to each well to solubilize the formazan. The absorbance at 570 nm (OD_570_–_630_) was measured using a Spectramax 250 microplate reader (BioTex, Bad Friedrichshall, Germany).

### 4.6. Preparation of Whole Cell Lysates and Nuclear Fractions for Immunoblot Analyses

The RAW264.7 cells (5 × 10^6^ cells/mL) were washed in cold PBS with 1 mM sodium orthovanadate and lysed using a sonicator (Thermo Fisher Scientific, Waltham, MA, USA) in lysis buffer for 30 min with rotation at 4 °C [39,40]. The lysates were clarified by centrifugation at 16,000× *g* for 10 min at 4 °C and stored at −20 °C until before the next experiments. The cell nuclear lysates were prepared in a three-step procedure, according to a previous method [41]. Following this treatment, the cells were harvested with a rubber policeman and washed with 1 × PBS. The cells were then lysed in 500 μL lysis buffers on ice for 4 min. Cell lysates were then spin-downed at 12,000 rpm for 1 min in a microcentrifuge. In the second step, the pellet (the nuclear fraction) was washed with the lysis buffer without Nonidet P-40. In the final step, the nuclei were treated with an extraction buffer (lysis buffer containing 500 mM KCl and 10% glycerol). The nuclei/extraction buffer mixture was kept to freeze at −80 °C and then thawed on ice and spin-downed at 19,300× *g* for 5 min. The supernatant was collected as the nuclear extract. Soluble cell lysates (30 μg/lane) were subjected to immunoblotting. The total levels of transcription factors (p65 and p50), protein kinase B (Akt), PDK1, p85, IκBα, IKKα/β, Syk, Src, Lamin A/C and β-actin were visualized as described previously [42]. The relative intensity of phospho-proteins was calculated using total-protein levels with the DNR Bio-imaging system (DNR Bio-Imaging Systems Ltd, Jerusalem, Israel).

RAW264.7 cells (5 × 10^6^ cells/mL) were washed three times in cold PBS containing 1 mM sodium orthovanadate and then lysed in lysis buffer (20 mM Tris-HCl, pH 7.4, 2 mM EDTA, 2 mM ethyleneglycotetraacetic acid, 50 mM β-glycerophosphate, 1 mM sodium orthovanadate, 1 mM dithiothreitol, 1% Triton X-100, 10% glycerol, 10 μg/mL aprotinin, 10 μg/mL pepstatin, 1 mM benzimide, and 2 mM PMSF) for 30 min with rotation at 4 °C. The lysates were clarified by centrifugation at 13,000× *g* for 10 min at 4 °C and stored at −20 °C until needed.

Nuclear lysates were prepared using a three-step procedure [43]. After treatment, the cells were collected with a rubber policeman, washed with 1 × PBS, and lysed in 500 μL lysis buffer containing 50 mM KCl, 0.5% Nonidet P-40, 25 mM HEPES (pH 7.8), 1 mM phenylmethylsulfonyl fluoride, 10 μg/mL leupeptin, 20 μg/mL aprotinin, and 100 μM 1,4-dithiothreitol (DTT) on ice for 4 min. The cell lysates were centrifuged at 13,000 rpm for 1 min in a microcentrifuge. Next, the pellet (the nuclear fraction) was washed once with wash buffer (lysis buffer without Nonidet P-40). The nuclei were treated with an extraction buffer containing 500 mM KCl, 10% glycerol, and several other reagents contained in the lysis buffer. Finally, the nuclei/extraction buffer mixture was frozen at −80 °C, thawed on ice, and centrifuged at 13,000 rpm for 5 min. The supernatant was collected as the nuclear extract.

For immunoblot analysis, proteins (25 μg/lane) were separated on 10% SDS-polyacrylamide gels and transferred to polyvinylidene difluoride (PVDF) membranes by electroblotting. Membranes were blocked for 60 min at room temperature in Tris-buffered saline containing 3% FBS, 20 mM NaF, 2 mM EDTA, and 0.2% Tween 20. The membranes were incubated for 60 min with specific primary antibodies at 4 °C, washed three times with the same buffer, and incubated for an additional 60 min with horseradish peroxidase (HRP)-conjugated secondary antibodies. The levels of total and phosphorylated inflammatory signaling and housekeeping proteins were visualized using an enhanced chemiluminescence (ECL) system (Amersham, Little Chalfont, Buckinghamshire, UK), as reported previously [44].

### 4.7. In Vitro Kinase Assay with Purified Enzymes

A kinase profiling service from Millipore (Billerica, MA, USA) was used to evaluate the inhibition of signaling kinases. Full-length purified Src, Syk, TAK1, IRAK1, or IRAK4 (1–5 mU) were incubated with reaction buffer in a final reaction volume of 25 μL. The reaction was initiated by the addition of MgATP (10 μM). After incubation for 40 min at room temperature, the reaction was stopped by the addition of 5 mL of 3% phosphoric acid solution. In addition, 10% of the reaction was spotted onto a P30 Filtermat (Fisher Scientific, Waltham, MA, USA) and washed three times for 5 min in 75 mM phosphoric acid and once in methanol prior to drying and scintillation counting.

### 4.8. Statistical Analyses

All data presented in this paper are expressed as the mean ± SD of experiments. For statistical comparisons, the results were analyzed using either ANOVA/Scheffe’s post hoc test or the Kruskal-Wallis/Mann-Whitney test. A *p* value < 0.05 was considered statistically significant. All statistical tests were carried out using the computer program SPSS (SPSS Inc., Chicago, IL, USA). Similar experimental data were obtained using an additional independent set of *in vivo* experiments conducted using the same number of mice.

## 5. Conclusions

**Figure 5:**
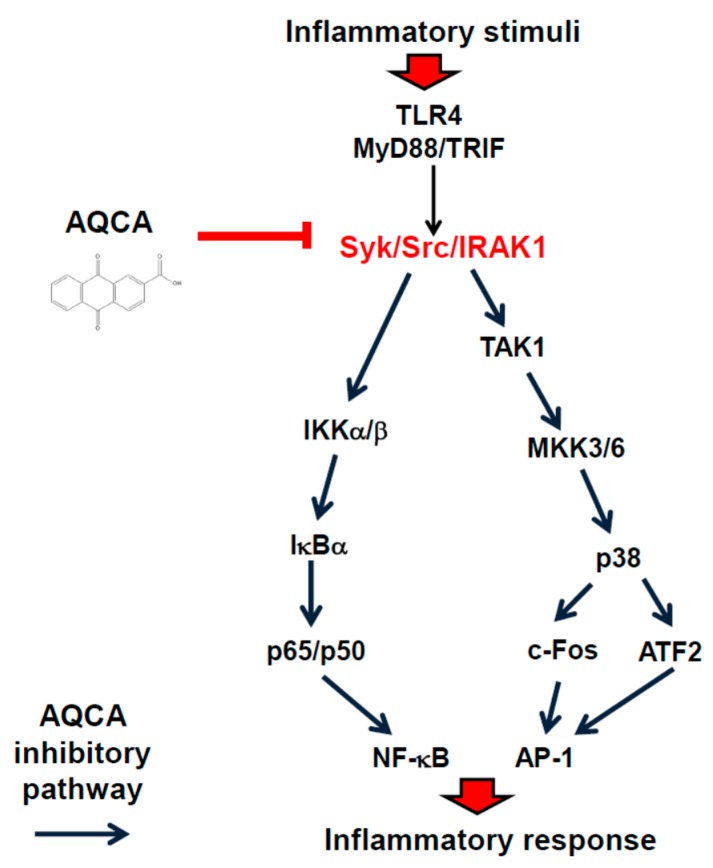
Putative pathway for the inhibition of inflammatory signaling events by AQCA in *in vitro* inflammatory responses.

In summary, the results of this study demonstrate that AQCA inhibits the signaling cascades of inflammatory responses without any cellular toxicity. AQCA downregulates the levels of inflammatory gene expression by inhibiting the activation of multiple enzymes including Src, Syk, p38, JNK, IRAK1, TAK1, and IRAK4, thus suppressing activation of NF-κB and AP-1, as summarized in Figure 5. Since AQCA is one of the major components of numerous vegetables, fruits, and medicinal herbs, we propose that AQCA or AQCA-rich fractions might be developed as functional foods or drugs with anti-inflammatory properties.

## Abbreviations

The following abbreviations are used in this manuscript:

NO	nitric oxide
MTT	(3-4,5-dimethylthiazol-2-yl)-2,5-diphenyltetrazolium bromide, tetrazole
AQCA	anthraquinone-2-carboxylic acid
2-HMAQ	2-hydroxymethylanthraquinone
PMA	phorbol-12-myristate
COX-2	cyclooxygenase-2
FBS	fetal bovine serum
iNOS	inducible nitric oxide synthase
IKK	IκB kinase
LPS	lipopolysaccharide
PAMPs	pathogen-associated molecular patterns
PBS	phosphate-buffered saline
PRRs	pattern recognition receptors
TLRs	toll-like receptors
TNF	tumor necrosis factor
Syk	spleen tyrosine kinase
IRAK4	interleukin-1 receptor-associated kinase 4
